# Baseline Clinical and Neuroimaging Biomarkers of Treatment Response to High-Frequency rTMS Over the Left DLPFC for Resistant Depression

**DOI:** 10.3389/fpsyt.2022.894473

**Published:** 2022-05-20

**Authors:** Ghina Harika-Germaneau, Issa Wassouf, Tom Le Tutour, Remy Guillevin, Damien Doolub, Reza Rostami, Alexia Delbreil, Nicolas Langbour, Nematollah Jaafari

**Affiliations:** ^1^Centre Hospitalier Henri Laborit, Unité de Recherche Clinique Pierre Deniker, Poitiers, France; ^2^Centre de Recherches sur la Cognition et l'Apprentissage, Centre National de la Recherche Scientifique (CNRS 7295), Université de Poitiers, Poitiers, France; ^3^Centre Hospitalier Nord Deux-Sèvres, Service de Psychiatrie Adulte, Thouars, France; ^4^CHU de Poitiers, Service de Radiologie, Poitiers, France; ^5^Laboratoire Dactim Mis, LMA, UMR CNRS 7348, Poitiers, France; ^6^Department of Psychology, University of Tehran, Tehran, Iran; ^7^Atieh Clinical Neuroscience Centre, Tehran, Iran; ^8^CHU Poitiers, Service de Médecine Légale, Poitiers, France

**Keywords:** rTMS (repetitive transcranial magnetic stimulation), major depression (MDD), bipolar disorder, structural MRI (sMRI), DLPFC (dorsolateral prefrontal cortex), high-frequency, response

## Abstract

**Background:**

Repetitive transcranial magnetic stimulation (rTMS) has proven to be an efficient treatment option for patients with treatment-resistant depression (TRD). However, the success rate of this method is still low, and the treatment outcome is unpredictable. The objective of this study was to explore clinical and structural neuroimaging factors as potential biomarkers of the efficacy of high-frequency (HF) rTMS (20 Hz) over the left dorso-lateral pre-frontal cortex (DLPFC).

**Methods:**

We analyzed the records of 131 patients with mood disorders who were treated with rTMS and were assessed at baseline at the end of the stimulation and at 1 month after the end of the treatment. The response is defined as a 50% decrease in the MADRS score between the first and the last assessment. Each of these patients underwent a T1 MRI scan of the brain, which was subsequently segmented with FreeSurfer. Whole-brain analyses [Query, Design, Estimate, Contrast (QDEC)] were conducted and corrected for multiple comparisons. Additionally, the responder status was also analyzed using binomial multivariate regression models. The explored variables were clinical and anatomical features of the rTMS target obtained from T1 MRI: target-scalp distance, DLPFC gray matter thickness, and various cortical measures of interest previously studied.

**Results:**

The results of a binomial multivariate regression model indicated that depression type (*p* = 0.025), gender (*p* = 0.010), and the severity of depression (*p* = 0.027) were found to be associated with response to rTMS. Additionally, the resistance stage showed a significant trend (*p* = 0.055). Whole-brain analyses on volume revealed that the average volume of the left part of the superior frontal and the caudal middle frontal regions is associated with the response status. Other MRI-based measures are not significantly associated with response to rTMS in our population.

**Conclusion:**

In this study, we investigated the clinical and neuroimaging biomarkers associated with responsiveness to high-frequency rTMS over the left DLPFC in a large sample of patients with TRD. Women, patients with bipolar depressive disorder (BDD), and patients who are less resistant to HF rTMS respond better. Responders present a lower volume of the left part of the superior frontal gyrus and the caudal middle frontal gyrus. These findings support further investigation into the use of clinical variables and structural MRI as possible biomarkers of rTMS treatment response.

## Introduction

Mood disorder [major depressive disorder (MDD) and bipolar disorder (BD)] is a heterogeneous and complex psychiatric condition. It is a major public health issue, ranking as the leading cause of disability worldwide, and the burden of mood disorders continues to grow despite the availability of validated interventions (1). MDD and BD both exhibit similar severe depressive symptoms (major depression, MD) (2).

The primary approaches to deal with MD include pharmacotherapy and psychotherapy. Although these approaches are effective, they still leave a significant proportion of patients with incomplete remission (3). This frequently results in TRD, which is associated with significant morbidity and high suicide risk (4).

As a result, several alternative treatments have been developed to target TRD, one of which is repetitive transcranial magnetic stimulation (rTMS) (5, 6). TMS is a non-invasive brain stimulation procedure that applies repeated magnetic pulses over the scalp to generate an electrical current in the cortex, provoking electrophysiological effects that modify the neural excitability in the target area and correlated brain networks (7). Safe profile (particularly the lack of systemic side effects associated with pharmacotherapy) and improved focality are some of its advantages over other neuromodulation techniques, such as electroconvulsive therapy (ECT).

Over 150 randomized controlled trials (RCTs) in unipolar and bipolar depression have been carried out, and their efficacy has been confirmed in multiple meta-analyses (8, 9). Moreover, real-world data have also confirmed the effectiveness of rTMS for major depressive disorder in clinical practice (10), with the most recent literature indicating response rates of 40–50% and remission rates of 25–30% (11).

The rTMS is effective in major depressive disorder but presents a high interindividual heterogeneity of clinical effectiveness (12). Moreover, this technique is costly in the real world and requires significant financial and time commitment from the patient and the practitioner. These elements highlight the pressing need for clinical and biological markers to predict treatment outcomes.

Clinical factors associated with rTMS response are divided into three main categories (13): patient-related factors (e.g., age, gender, and treatment resistance); illness-related factors (e.g., bipolar depression, duration and severity of depression, therapeutic resistance, previous response to rTMS or ECT), and TMS procedure-related factors (e.g., TMS intensity, number of pulses per session, and number of sessions) (14). Despite the extensive literature, data remain heterogeneous and contradictory with the need to be pursued.

Recent studies suggest that neuroimaging markers may achieve higher predictive accuracy than clinical or demographic variables [for review, see (15)]. Earlier studies showed promising results using methods derived from resting-state functional MRI or diffusion-weighted MRI (16). However, these biomarkers involve complex imaging protocols with few patients and a very specific patient selection. These imaging methods are rarely available in clinical settings, need specialized data processing, and are costly. Therefore, as suggested by Baeken et al., (17), simpler biomarkers like cortical thickness measures, as derived from anatomical MRI data, could be more feasible in current clinical practice. Indeed, to date, two studies have explored cortical thickness before stimulation as a predictor of rTMS response in patients with MDD (17, 18).

We conducted a retrospective naturalistic study to evaluate whether clinical factors or cerebral cortex thickness and volume may be a potential biomarker of rTMS treatment response in drug-resistant patients with depression of a large dataset from patients that received rTMS for the treatment of depression in a real-world clinical setting of a tertiary referral hospital.

## Materials and Methods

Between September 2014 and February 2018, we revised and analyzed the records of 131 patients with mood disorders who received rTMS treatment in the Neurostimulation Department of Henri Laborit Psychiatric Hospital. Each patient was treated only one time with rTMS. Non-opposition to the use of the participants' research data was obtained retrospectively. All patients provided informed written consent, and the study was registered at the Health Data Hub platform (F20210128152411).

### Patients and Assessment

Patients treated with rTMS in our department met the criteria for major depressive disorder or bipolar disorder as defined by DSM-IV-TR. The diagnosis was made using the Mini International Interview for Neuropsychiatric Disorders (19) by an experienced psychiatrist. All patients had to be in a current major depressive episode with a MADRS score higher than 20. The exclusion criteria included a DSM-IV-TR Axis I of psychotic disorder, a DSM-IV-TR diagnosis of alcohol or substance dependence, significant current active medical problem, and known neurological disease or a contraindication to rTMS (e.g., history of seizure disorder, presence of a pacemaker or metal somewhere in the head other than in the teeth) or MRI scanning (aneurysm clips, stents, or metal anywhere in the body).

Treatment resistance was defined as non-responsiveness to at least two courses of antidepressant medications for at least 6 weeks [Stage II, of Thase and Rush's definition (20)], as determined by their primary treating clinician and patient judgment of medication effectiveness. No medications changes were allowed in the 3 weeks before the beginning of the rTMS treatment or during the rTMS treatment itself.

The inclusion criteria for the retrospective analysis were as follows: rTMS-naïve (only the patient's first treatment with rTMS was considered), primary diagnosis of a depressive disorder (including bipolar disorder, currently depressive episode, major depressive disorder, and recurrent depressive disorder), a complete documented MADRS at the beginning (baseline), at the end of rTMS treatment (Day 14), and 1 month after (Day 45) the end of the rTMS course, and absence of a serious somatic illness. Both in- and out-patients were included.

Trained psychiatrists completed clinical assessments. All assessments included MADRS and BDI. Patients were assessed at baseline, post rTMS treatment (after 2 weeks of treatment, D14), and at one-month follow-up (45 days after baseline, D45). The primary outcome measure was the total MADRS score. The responder status was defined as a 50 % decrease in the MADRS score.

### Treatment

Before the treatment period, each patient underwent an anatomical T1-weighted magnetic resonance imaging to set up the neuronavigation system (Syneika One; Syneika). The left DLPFC is detected by the Syneika neuronavigation system, which uses T1 imaging and the Talairach atlas to define the optimal target. The following acquisition parameters were used: Axial 3D T1 MPRAGE: TR, TE, TI = (2,000, 2.54, 900) ms; slice thickness 0.799 mm, Nex 1. The coil was positioned to target the left DLPFC. All patients had their motor cortex excitability evaluated at baseline and weekly using the Resting Motor Threshold (RMT). For 2 weeks, 10 rTMS sessions were delivered one time daily, five days a week. The rTMS treatment was administered with the MagPro^®^ X100 with an option stimulator (MagVenture, Inc) using the Figure 8 coil.

Stimulation parameters were as follows: stimulation intensity was 110% of resting RMT, the stimulation frequency was 20 Hz, the train duration was 2 s, the inter-train interval was 10 s, the number of trains per session was 80, and the total number of pulses per session was 3,200. The stimulation lasted approximately a quarter of an hour (16 min) (21, 22). The rTMS protocol is based on the French guidelines. The variables for frequency and train duration were based on the study of Machii et al. (21), for the inter-train interval on the study of Chen et al. (22), and for the number of pulses on the study of Naihaus et al. (23). The number of sessions was determined by the previous trial with a frequency of 20 Hz (24, 25).

### Anatomical Measures

The DICOM MRI images were converted to the Nifti format with a 1 mm isometric spacing and were used as input to FreeSurfer 6 software to compute the segmentation of white matter and cortical regions defined in the Desikan–Killiany atlas (26). This was accomplished by running brain pictures through the “recon-all” pipeline, which consists of skull-stripping, segmentation of gray and white matter voxels, tessellation, inflation, and registration to a brain template. In the previously mentioned atlas, the left-DLPFC will be defined as the union of the superior frontal and the caudal and the rostral middle frontal gyri. These three structures were then truncated at Talairach coordinates, at *y* = 26 [similar to Ehrlich et al. (27)] to filter out the pre-motor areas and at *x* = −15 to get rid of the medial regions. Cortical volume and thickness were then extracted for each subject within this region using the *mris_anatomical_stats* command. Other parameters will be studied, as they have previously been highlighted for their link with clinical response (28): left and right hippocampus volumes, left and right amygdala volumes, and left and right ACC, which are obtained by adding the volumes of caudal and rostral anterior cingulate as defined in the Desikan-Killiany atlas. The volumes of the hippocampus and the amygdala were obtained using the *asegstats2table* command, while the volumes of rostral-ACC and caudal-ACC were obtained using the *aparcstats2table* command. All volumes are divided by the estimated total intracranial volume (eT as computed by FreeSurfer.

After registering all patient segmentations to an average space, a whole-brain analysis was performed using the FreeSurfer tool QDEC (“Query, Design, Estimate, Contrast”). The null hypothesis states that the two groups' intercepts are not significantly different, which is equivalent to checking whether the average measure at a given vertex differs significantly between responders and non-responders. These results were then corrected for multiple observations using a Montecarlo null-Z simulation with a significance threshold of at least 0.05. Results that did not pass corrections were not considered. Following the literature, the analysis was carried out for the measures of thicknesses and volumes on the left and right hemispheres with a smoothing kernel of 15 mm (17, 18). We added the clinical variables as nuisance factors if their *p*-value in the univariate tests was inferior to 0.2. However, age and gender were added as nuisance factors regardless.

### RTMS Targeted Anatomical Features

**Figure 1:**
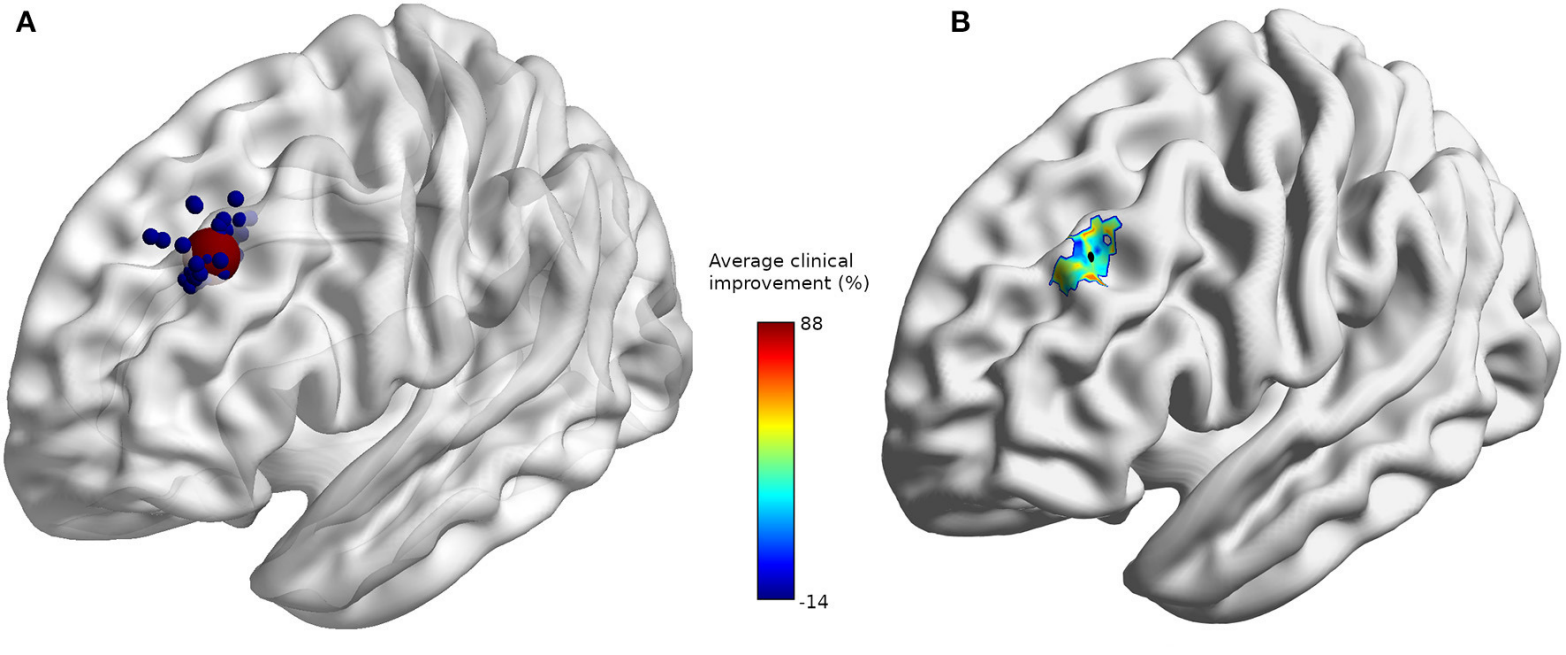
**(A)** Individual stimulation sites (blue) and average stimulation site (red). **(B)** Average clinical improvement (%) with average stimulation site (black) at coordinates [−32, 32, 38].

The coordinates of the individual stimulation targets for all subjects were extracted. pheres with a radius of 2 mm were created for each stimulation target using the SPM add-on MarsBar (29) and overlaid on an average brain using BrainNet Viewer (30) (Figure 1A). A sphere with a 10 mm radius was defined as the mean position target (red dot) of all subjects' coordinates. The mean sphere of interest will be used to extract anatomical features of TMS. To provide additional information, each subject's individual target coordinates were weighted according to their clinical improvement, and the mean improvement field was displayed on an average brain (Figure 1B) (29). The minimum brain-scalp distance between the target and the scalp was computed using the freely available ScalpGM tool (31)^1^, which relies on the SPM toolbox (spm12)^2^ ScalpGM performs segmentation, computes minimal scalp distance for each gray matter voxel, and then warps the distance maps to a common space (MNI) for comparison. We used the mean radius sphere to extract scalp distance for all subjects. ScalpGM maps were thresholded at 1 mm (we assume that any value below 1 mm could not possibly be picked up since it would be inferior to the original image spacing and was therefore discarded as an artifact). To preserve the original image range, the interpolation type was set to the nearest neighbors. The cortical distance was defined as the minimal distance exceeding a threshold of 1 mm in the limits of the mean sphere.

### Statistical Analysis

We used jamovi software (The jamovi project (2021). jamovi (Version 2.2.3.0). Retrieved from^3^) to conduct univariate statistical tests. The responder status was analyzed using binomial multivariate regression models. First, using clinical variables (gender, age, type of depression, resistance stage, baseline MADRS score, duration of illness, and current episode). Second, rTMS target anatomical features (target minimal distance and gray matter thickness at target) were added. Finally, the specific volumes of interest, such as left and right hippocampal volumes, left and right amygdala volumes, left and right ACC volumes, and left and right insula, that were previously highlighted for their link with clinical response were added The clinical covariates were included in the whole-brain analysis using the same method as the one used for the nuisance factors (univariate model *p* <0.2; gender and age always included). Statistical significance is set at *p* < 0.05; a *p* <0.1 will be considered a noteworthy trend. Additionally, robust binomial multivariate regressions were performed to mitigate the impact of the non-normality of the data. The implementation used is the *glmrob* function implemented in the *robustbase* R package (32). The results presented will be of the non-robust multivariate binomial model.

## Results

All patients in our study population were taking antidepressants and/or thymoregulators, with 3.2% on lithium and 22.1% on anti-epileptic drugs prescribed for thymoregulatory purposes (clonazepam, lamotrigine, oxcarbazepine, and valpromide), with the exception of pregabalin, which was prescribed as an anxiolytic. The majority of patients were treated with one or more antidepressants: 40% were on SSRIs, 27.4% on SNRIs, 24.2% on tricyclic antidepressants, 5.3% on tetracyclic antidepressants, 3.2 % on MonoAmine Oxidase Inhibitors (MAOI), and 8.4 % were prescribed other types of antidepressants. Benzodiazepines or related drugs were prescribed for a majority of patients (80%) and antipsychotics for approximately half of the patients (53.7%).

Of the 131 patients, 20 refused to participate in the study. The final assessment of the MADRS score was missing for 16 patients. For one patient, the MADRS assessment on day 14 was missing. For 94 patients with neither of these variables missing, the evolution between day MADRS0 and MADRS14, as well as the evolution of MADRS0 and MADRS45, was computed and found to be moderately correlated (Pearson's r, r = 0.475, *p* < 0.001). As a result, we decided to exclude from the following analyses any patient whose final assessment was missing. We, therefore, included 95 patients. Some clinical factors, such as depression type (*p* = 0.034), sex (*p* = 0.022), and Thase and Rush resistance stage (*p* = 0.053), play an important role (Table 1). Table 2 highlights the differences between unipolar and bipolar patients.

**Table 1:** Clinical factors and univariate tests.

**Variable**	**N**	**NR**	**R**	**Test statistic**	***p*** **-value**
Age	95 (NR = 57; R = 38)	52.12 ± 11.59	51.45 ± 10.75	U = 1,038.50**	0.738
Duration of the episode	94(NR = 57; R = 37)	38.76 ± 46.12	38.08 ± 57.83	U = 1,009.00**	0.728
Duration of illness	91 (NR = 56; R = 35)	184.39 ± 151.00	225.16 ± 148.85	U = 794.00**	0.130
Baseline Beck score	91 (NR = 56; R = 35)	20.11 ± 6.32	19.09 ± 0.66	U = 908.00**	0.559
Baseline MADRS score	95 (NR=57; R = 38)	28.91 ± 5.39	27.50 ± 5.63	U = 899.00**	0.162
Resistance stage	95 (NR = 57; R = 38)	2.65 ± 0.94	2.32 ± 0.66	U = 847.00**	0.053
Motor Threshold	95 (NR = 57; R=38)	51.11 ± 9.07	53.29 ± 9.35	U = 965.50**	0.374
Depression type: Bipolar	95 (NR = 57; R = 38)	10/57	14/38	X^2^ (1) = 4.50*	0.034
Gender: F	95 (NR = 57; R = 38)	27/57	27/38	X^2^ (1) = 5.21*	0.022
SSRIs	95 (NR = 57; R = 38)	22/57	16/38	X^2^ (1) = 0.117*	0.732
SNRIs	95 (NR = 57; R = 38)	16/57	10/38	X^2^ (1) = 0.0353*	0.851
Tricyclic antidepressants	95 (NR = 57; R = 38)	13/57	10/38	X^2^ (1) = 0.153*	0.696
Tetracyclic antidepressants	95 (NR = 57; R = 38)	3/57	2/38	X^2^ (1) = 0.000***	1.000
MAOIs	95 (NR = 57; R = 38)	3/57	0/38	X^2^ (1) = 0.702***	0.402
Other	95 (NR = 57; R = 38)	4/57	4/38	X^2^ (1) = 0.0512*	0.821
Benzodiazepine	95 (NR = 57; R = 38)	45/57	31/38	X^2^ (1) = 0.0987*	0.753
Anti-epileptics	95 (NR = 57; R=38)	12/57	9/38	X^2^ (1) = 0.0917*	0.762
Anti-psychotics	95 (NR = 57; R = 38)	33/57	18/38	X^2^ (1) = 1.0160*	0.313
Lithium	95 (NR = 57; R = 38)	1/57	2/38	X^2^ (1) = 0.129***	0.719

*Pearson's X^2^; **Mann–Whitney U; ***Continuity-corrected X^2^. NR, Non-Responders on Day 45; R, Responders on Day 45.

**Table 2:** Differences in clinical factors and MADRS scores between patients with unipolar disorder and bipolar disorder.

**Variable**	***N***	**Unipolar**	**Bipolar**	**Test statistic**	***p*** **-value**
Age	95 (Unipolar = 71; Bipolar = 24)	52.32 ± 11.08	50.46 ± 11.72	U = 783**	0.557
Gender: F	95 (Unipolar = 71; Bipolar = 24)	40/71	14/10	X^2^ (1) = 0.0291*	0.865
Benzodiazepine	95 (Unipolar = 71; Bipolar = 24)	56/71	20/24	X^2^ (1) = 0.0314***	0.859
Baseline MADRS score	95 (Unipolar = 71; Bipolar = 24)	28.80 ± 5.61	27.00 ±5.03	U = 678**	0.136
Day 14 MADRS score	95 (Unipolar = 71; Bipolar = 24)	20.73 ± 9.21	13.50 ± 8.74	U = 477**	0.001
Day 45 MADRS score	95 (Unipolar = 71; Bipolar = 24)	19.75 ± 9.01	13.13 ± 8.78	U = 510**	0.003
Day 14 Responder	95 (Unipolar = 71; Bipolar = 24)	20/71	13/24	X^2^ (1) = 5.35*	0.021
Day 45 Responder	95 (Unipolar = 71; Bipolar = 24)	24/71	14/24	X^2^ (1) = 4.50*	0.034

*Pearson's X^2^; **Mann–Whitney U; ***Continuity-corrected X^2^.

If the duration of illness or current episodes was missing from subsequent analyses, it was replaced by the mean of the entire population. Five additional patients were excluded due to a failed parcellation or a faulty MRI acquisition, resulting in a sample size of 90 subjects for the analyses relying on MRI data.

None of the patients participating in the study reported having seizures or shifting between hypomanic/manic states. Patients did not complain about local pain or dizziness after stimulation. Moreover, none of the patients discontinued treatment due to adverse effects.

### Whole-Brain Analysis

**Figure 2:**
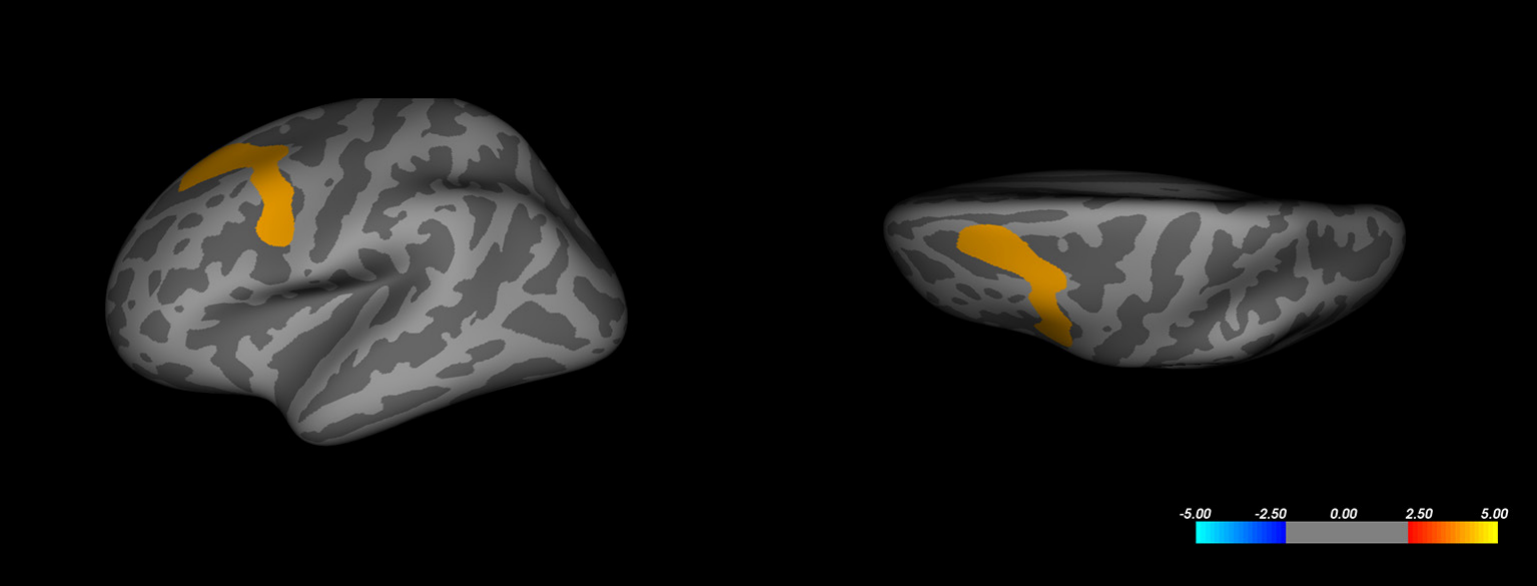
Whole-brain analysis corrected cluster on left hemisphere cortical volume. Color represents significance as a Z-score. Corrected maximal significance Z = 4.000 is at Talairach coordinates [−30.8, 9.3, 53.6] (FreeSurfer annotation: caudal-middle frontal).

Whole-brain analysis based on the volume difference between responders and non-responders (comparing the intercepts of the two groups) revealed a decreased volume for the responder's group in the superior-frontal and caudal middle frontal regions of the left hemisphere (Figure 2). This result passed the Monte-Carlo null-Z cluster correction up to a threshold of 0.005 (Z-score of 2.3). The same observation could not be reproduced in the opposite hemisphere. No significant difference was observed in cortical thickness between the responder and non-responder groups, even at a more relaxed correction threshold of 0.05 (Z-score of 1.3).

### Region of Interest Analyses

Gender, type of depression, and resistance stage all appear to play a role in the binomial model containing the DLPFC measures (Table 3), consistent with the results of the univariate tests conducted previously. The baseline MADRS score seems to be significant as well (*p* = 0.027). Imagery-based measures, such as the left DLPFC thickness and the minimal scalp distance to the cortical target, did not appear to be significant (both *p* > 0.1).

**Table 3:** Multivariate binomial regression coefficients of DLPFC measures and clinical factors.

**Predictor**	**Estimate**	**SE**	**Z**	***p***	**Odds ratio**
Intercept	0.440149	4.531280	0.097136	0.923	1.550
Gender: M – F	−1.454878	0.565119	−2.574462	0.010	0.230
Depression type: Unipolar – Bipolar	−1.380683	0.616408	−2.239885	0.025	0.250
Age	0.023513	0.026489	0.887671	0.375	1.020
Resistance stage	−0.690661	0.359458	−1.921395	0.055	0.500
Duration of illness	0.000000	0.001897	0.000030	1.000	1.000
Baseline MADRS score	−0.117649	0.053201	−2.211380	0.027	0.890
DLPFC average thickness	2.361551	1.467189	1.609575	0.107	10.610
Minimal brain-scalp distance	−0.053008	0.037102	−1.428713	0.153	0.950

*Deviance = 97.095; R^2^ [Nagelerke's] = 0.31697; χ^2^ = 24.047; df = 8; p = 0.002; accuracy = 0.7; specificity = 0.74074; sensitivity = 0.63889; AUC = 0.78601*.

The second model (Table 4) does not appear to demonstrate the significance of any of the volumes considered (all *p* > 0.1). Gender, type of depression, stage of resistance, and severity of depression represented by the baseline MADRS score retain their significance. Clinical factors appear to have the greatest influence on predicting individual treatment responses in both models, owing to the low variation of the model fit and predictive measures.

**Table 4:** Multivariate binomial regression coefficients of literature volumes and clinical factors.

**Predictor**	**Estimate**	**SE**	**Z**	**p**	**Odds ratio**
Intercept	6.787604	4.589525	1.478934	0.139	886.786
Gender: M – F	−1.625463	0.628189	−2.587536	0.010	0.197
Depression type: Unipolar – Bipolar	−1.470419	0.682712	−2.153790	0.031	0.230
Age	0.004934	0.029839	0.165357	0.869	1.005
Resistance_stage	−0.764139	0.381127	−2.004945	0.045	0.466
Duration_of_illness	−3.3961*e*−4	0.001926	−0.176332	0.860	1.000
MADRS_J0	−0.119086	0.057000	−2.089244	0.037	0.888
Left_Amygdala	21.738405	35.078504	0.619707	0.535	2,759,700,000.000
Right_Amygdala	4.182690	31.611853	0.132314	0.895	65.542
Left_Hippocampus	4.585550	18.287139	0.250753	0.802	98.057
Right_Hippocampus	−11.560154	18.888773	−0.612012	0.541	0.000
lh_insula_volume	−6.102632	7.858057	−0.776608	0.437	0.002
rh_insula_volume	0.229891	7.950897	0.028914	0.977	1.258
lh_ACC_volume	7.054542	7.553866	0.933898	0.350	1158.106
rh_ACC_volume	6.737072	6.511928	1.034574	0.301	843.089
DLPFC_volume	−2.444911	2.623072	−0.932079	0.35	0.087

*Deviance = 96.831; R^2^ [Nagelerke's] = 0.3200; χ^2^ = 24.311; df = 15; p = 0.06; accuracy = 0.74444; specificity = 0.81481; sensitivity = 0.63889; AUC = 0.78704*.

The results of the robust analyses did not appear to change drastically. The robust model for literature volumes showed a decrease in the significance of depression type (from significant to *p* < 0.1) and in the resistance stage (*p* < 0.1 to insignificant). On the model containing the DLPFC measures, no such changes were observed.

## Discussion

In this retrospective and naturalistic study, we aimed to identify clinical and neuroimaging factors associated with the efficacy of rTMS evaluated 1 month after the beginning of treatment in 95 patients with drug-resistant depression who were treated with high-frequency rTMS (20 Hz) over the left DLPFC.

The response rate at 1 month after rTMS treatment in our study is 40%. This is consistent with previous studies reporting a 40–50% response rate to rTMS treatment in MDD (33, 34) and BDD (35–37).

Our analysis of clinical variables (type of depression, illness severity evaluated by MADRS and Beck, resistance stage, associated treatment, gender, and resting state) demonstrated that the type of depression, gender, and resistance stage are associated variables with response to rTMS.

First, we found that bipolar depression responds better to HF rTMS over the left DLPFC. To our knowledge, no naturalistic study to date has highlighted bipolar illness as a factor in better response to rTMS or has established a correlation between the type of depression and the clinical effect of rTMS (12, 38). Treating bipolar depression is clinically challenging as antidepressants can worsen the outcome for this category of patients, which is why rTMS has been suggested as a treatment option for bipolar depression (39). Patients with bipolar depression were enrolled in studies focused primarily on unipolar major depression or in dedicated sham-controlled studies examining the efficacy of rTMS in bipolar depression. Three meta-analyses and quantitative syntheses have been conducted to date (9, 40, 41). According to Nguyen et al., active-rTMS is associated with a higher response rate than sham-rTMS in bipolar depression, but subgroup analyses testing differences based on stimulation target and site revealed no significant differences. However, when analyzed separately, HF over the left DLPFC stimulation was associated with a statistically significant greater response than sham treatment. In contrast, bilateral stimulation and low-frequency rTMS over the right DLPFC were not.

We identified the effectiveness of rTMS in bipolar depression in our study. This result differs from that of Yang et al. (42). In fact, in their naturalistic study with an adequately large cohort of participants, they suggest that patients with BD are less likely to achieve clinical response with high-frequency L-DLPFC rTMS than those with unipolar depression (10 Hz). The antidepressant response to rTMS might vary with stimulation frequency. In our study, we used 20 Hz stimulation in seven sham-controlled studies (43–49) in patients with mixed depression and one naturalistic study in patients with BDD (50). Apart from the same frequency of stimulation, there is still significant methodological heterogeneity between studies, including trial duration, stimulation intensity, and several pulses per session, which makes it difficult to compare different findings. Therefore, the stimulation parameters used in our study could account for the improved response in patients with BDD. This difference in response to treatment could be explained by differences in the clinical expression of bipolar and unipolar depressive episodes (51) and indeed by the potential differences in the neurophysiological mechanisms that cause them.

Second, in our study, patients with a high treatment resistance stage respond less to rTMS. The refractoriness of depressive episodes appears to be one of the best-supported predictors of rTMS response. Many studies have suggested that a higher degree of medication resistance may be tied to worse rTMS outcomes in depression (13). Most findings from rTMS response predictor studies suggest that a lower degree of drug resistance is one of the more robust predictors of superior outcomes for rTMS therapy using standard stimulation parameters and targeting methods. While definitive prospective studies are still needed, the existing literature appears to support the use of standard rTMS therapy relatively early during the treatment, prior to the occurrence of numerous medication treatment failures.

Third, we found that women respond better to rTMS than men do. The association between female gender and response to treatment is debatable. A recent meta-analysis, which included 54 sham-controlled trials between 1997 and 2013, revealed that gender might be a positive predictor of response, as studies showing good antidepressant response to rTMS had more female patients (52). In fact, in our study, women (56.8 %) outnumber men (43.2 %). In a second meta-analysis, the same authors show that the antidepressant effect of specifically HF rTMS was higher in RCTs with a greater proportion of female patients (53). They suggested that women's profiles, rather than their sex, might have influenced their response to treatment.

Other clinical and demographic variables, such as age, associated treatment, and resting motor threshold, had no effect on treatment response in our study. The link between treatment response and these variables is debated in the literature. Some studies revealed that younger patients respond better to rTMS (54, 55), whereas other studies found no correlation between age and response (56). Regarding associated treatment, there is growing evidence that concomitant use of medication can impair the clinical effectiveness of rTMS, especially for benzodiazepines (57). However, the impact of concomitant medication on rTMS effectiveness is still debatable (58, 59). Finally, we did not find a link between baseline RMT and response. The correlation between clinical efficiency and stimulation intensity is not precisely known (60, 61). Some studies suggest a dose efficiency correlation. Another hypothesis assumes a more complex and non-linear correlation.

We also investigated the relationship between structural neuroimaging variables (whole-brain analysis, thickness of the left DLPFC and ACC, volumes described in the literature, and distance between scalp and cortical target) and response to rTMS. Neuroanatomical predictors may be particularly useful for brain stimulation interventions, which directly modify the activity within neural circuits, contrary to indirect reorganization caused by psychological or pharmacological interventions.

We initially performed an exploratory analysis of thickness and volume across the brain, in addition to the main cortex-wide analysis that was unconstrained by a priori hypotheses. We also performed ROI analyses to further evaluate the *a priori* hypotheses and then explored specifically the link between the left DLPFC and rostral ACC thickness at baseline and treatment response.

We did not find a statistically significant link between cortical thickness and treatment response. The link between cortical thickness and the responses to ECT (62), tDCS (63), and rTMS (17, 18) has been reported before. Boes et al. (18) describe that cortical thickness in the left rostral anterior cingulate cortex region correlates with rTMS treatment response in 48 TRD patients treated with HF (10 Hz) rTMS over the left DLPFC baseline. In fact, patients with thinner cortex before treatment tended to have the most clinical improvement. Baeken et al. (17) recently suggested that baseline cortical thickness in the right caudal part of the anterior cingulate cortex was significantly correlated with direct clinical responses in the subgroup that received active aiTBS (21 patients) over the left DLPFC during the first stimulation week, but no correlation was found with delayed response. In this study, we did not confirm the results of these two preceding studies. In Boes and Baeken study's, no accurate correction for multiple comparison testing was performed, increasing the risk of type I error. This can partly explain why we did not find the same results. Different stimulation parameters and the number of patients are also potential explanations for this discrepancy. Moreover, increased cortical thickness after rTMS treatment has been described in longitudinal studies (18, 64).

Response to rTMS was also evaluated in terms of the cortex-scalp distance. According to Lee et al. (65), the non-invasive brain stimulation scalp-to-cortex distance has been reported to critically influence the focality and strength of the electric field induced by rTMS. Our result indicated that there is no difference inefficacy related to this distance. Kozel et al. (66) discovered that these distances do not directly correlate with antidepressant clinical response in 29 depressed patients, but a correlation was established between the motor threshold measurement and the distance from the cortex to the skull under the TMS coil. In our study, the scalp-motor cortex distance was not possible because the stimulation point was not recorded during each session. Moreover, the lack of statistical correlation between response and distance in our study could have been influenced by the position of the stimulation coil. This could be in part due to the fact that the exact targeting zone for every session is unknown. One could also question the validity of the chosen region of interest (10 mm radius sphere around the average theoretical cortical target.

Gray matter volume (GMV) at baseline has been previously described as a predictor of treatment response in mood disorders. We first performed a whole-brain mapping without an *a priori* hypothesis for a specific brain volume. Interestingly, when taking into account depression type, gender, age, resistance stage, duration of illness and episode, and baseline MADRS score, this whole-brain analysis associated with clinical factors in a regression model shows that the average volume of the left part of the superior frontal gyrus and the caudal middle frontal gyrus was associated with the status of the response, where responders present a lower gray matter volume. The same result could not be reproduced in the right hemisphere.

The superior frontal gyrus and the middle frontal gyrus are usually defined as a part of the DLPFC (67). The superior frontal gyrus contributes to higher cognitive functions (68). It is part of the “hate circuit,” which is involved the pathogenesis of depression symptoms, risk and action responses, attention, reward, and emotion (69). The middle frontal gyrus is critical for higher-order executive functions related to stress perception and appraisal, including attention, working memory, planning, executive cognition, and emotion regulation (70–72), and may confer vulnerability to depression. The GMV deficits of the frontal cortex have been reported in several studies on MDD. Abe et al. (16) found that patients with MDD might have GMV deficits in frontal-temporal-limbic regions, which also included the middle frontal gyrus. Leung et al. (73) found that attention biases toward negative stimuli are associated with a reduced gray-matter concentration in the right superior frontal gyrus. Lai et al. (74) found a GMV increase in the frontal lobe after treatment with aripiprazole in patients with depression and deficits in the superior and medial frontal gyrus for patients with MDD at baseline status. Moreover, Lai et al. (74) compared structural differences between patients who were able to achieve remission and those who responded poorly to antidepressants. The remitting MDD patients showed a bilaterally smaller superior frontal gyrus volume. Yuan et al. (75) found that geriatric patients with depression in remission from their first episode of depression had reduced GMV in the right superior frontal gyrus in comparison with well-matched healthy controls. Although the nature of the involvement of the superior frontal gyrus and the caudal middle frontal gyrus in mood disorders remains a matter of debate, in our study, a greater volume in the left part of the superior and the caudal middle frontal gyrus was observed in non-responder patients. The association of this region with response to rTMS was not previously described in structural MRI studies that investigated response factors.

No statistically significant differences in baseline structural volumes were found between treatment responders and non-responders. Few studies have investigated the association between GMV and treatment response in MDD. Treatment response was evaluated for cognitive behavioral therapy (CBT) (76), antidepressant (77), ECT (78, 79), and rTMS (80). The study found a link between clinical response and the volumes of the left and right hippocampus, the left and right amygdala, the left and right ACC, and the left and right insula (28). In our study, none of these regions were found to be associated with response to rTMS.

Despite the strengths of this study (larger number of patients than already described in the literature, correction for multiple comparison testing with independent logistic regression models, naturalistic design), several limitations must be considered. First, all of the patients included in this study were under medication; prior exposure to medication is a strong confounding factor as it may affect the brain structure. In fact, Hoexter et al. (81) found that the thickness of the orbitofrontal cortex in patients with OCD can serve as a predictive biomarker of treatment response, exclusively in treatment-naive patients. Second, our investigation does not have a placebo-controlled group, which means that any predictive biomarker of treatment response could be confounded by the placebo effect. Third, our patients have multiple comorbidities, which may have confounded our results. Finally, MRI was performed only at baseline. In the future, recording structural MRI data at multiple time points to retrieve information about structural changes after rTMS is recommended.

There remains significant interest in understanding how to optimize the application of rTMS for each patient in order to achieve greater remission rates and provide more efficient symptom relief. The use of structural MRI is an essential tool to achieve this objective. Besides, this type of imaging is easy to perform, and collaborations between several centers can be envisioned to allow for the acquisition of a sufficient volume of imaging and clinical data in the future to establish solid correlations. Moreover, it would be interesting to characterize the structural covariance networks (SCN) to better understand the response to rTMS. SCN analyses aim to identify network patterns of common influences and characterizations within the brain across the population rather than differences in the structure of isolated brain regions in individuals (82). In addition, MDD is associated with deregulation of neural networks rather than a disruption of individual brain regions in isolation (80). Recently, preliminary evidence suggested that gray matter could be used to distinguish rTMS responders and non-responders, particularly in the fronto-parietal network (83, 84).

## Conclusion

In conclusion, we investigated the clinical and neuroimaging biomarker associated with the response to high-frequency rTMS over the left DLPFC in a large sample of patients with TRD depression. Women, patients with BDD, and patients who are less resistant were found to respond better to HF rTMS. Responders present a lower volume of the left part of the superior frontal and the caudal middle frontal gyri. The thickness of the DLPFC and ACC, the volumes of the amygdala, hippocampus, ACC, insula, DLPFC, and the distance from the scalp to the target were not associated with the clinical response. Our results reinforce the need to identify accurate and reliable clinical and neuroimaging biomarkers of treatment response. This biomarker that can be translated into clinical practice holds promise for the advancement of precision medicine. Our findings may serve as a guide to future studies with larger datasets to investigate specific neuroimaging biomarkers (the distance between scalp and target and specific volume) and clinical biomarkers (sociodemographic and clinical characteristics), with the ultimate aim of defining a multimodal biomarker profile that predicts rTMS treatment response.

## Data Availability Statement

The raw data supporting the conclusions of this article will be made available by the authors, without undue reservation.

## Ethics Statement

Ethical review and approval was not required for the study on human participants in accordance with the local legislation and institutional requirements. Written informed consent for participation was not required for this study in accordance with the national legislation and the institutional requirements.

## Author Contributions

GH-G, IW, DD, and NJ conducted the patient assessments. RG provided MRI images of the patients. GH-G, NL, and TL and performed analyses. The manuscript was written by GH-G and TL and all authors revised and proofread the manuscript.

## Funding

This study was supported by recurrent internal funds from the Center Hospitalier Henri Laborit, Poitiers, France.

## Conflict of Interest

The authors declare that the research was conducted in the absence of any commercial or financial relationships that could be construed as a potential conflict of interest.

## Publisher's Note

All claims expressed in this article are solely those of the authors and do not necessarily represent those of their affiliated organizations, or those of the publisher, the editors and the reviewers. Any product that may be evaluated in this article, or claim that may be made by its manufacturer, is not guaranteed or endorsed by the publisher.

## Footnotes

^1^ https://github.com/nickjdavis/ScalpGM

^2^ https://www.fil.ion.ucl.ac.uk/spm/software/spm12/

^3^ https://www.jamovi.org
